# Soft and Hard Tissue Grafting in Immediate Implant Therapy: A Narrative Review

**DOI:** 10.3390/medicina61101769

**Published:** 2025-09-30

**Authors:** Carlos A. Jurado, Fabio Andretti, Gerardo Guzman-Perez, Mark Adam Antal, Silvia Rojas-Rueda, Franciele Floriani, Kelvin I. Afrashtehfar, Nicholas G. Fischer

**Affiliations:** 1 Division of Operative Dentistry, Department of General Dentistry, College of Dentistry, The University of Tennessee Health Science Center, Memphis, TN 38104, USA; 2 School of Dentistry, Ponce Health Sciences University, Ponce 00716, Puerto Rico; 3 Periodontics Residency Program, Multidisciplinary Educational Center in Oral Rehabilitation (CEMRO), Morelia 58880, Mexico; 4 Department of Operative and Esthetic Dentistry, Faculty of Dentistry, University of Szeged, 6720 Szeged, Hungary; 5 Division of Dental Biomaterials, Department of Clinical and Community Sciences, School of Dentistry, University of Alabama at Birmingham, Birmingham, AL 35233, USA; 6 Department of Prosthodontics, College of Dentistry and Dental Clinics, The University of Iowa, Iowa City, IA 52242, USA; 7 Department of Reconstructive Dentistry and Gerodontology, School of Dental Medicine, University of Bern, 3010 Bern, Switzerland; 8 Oral Implantology Research Institute (OIRI), Dubai P.O. Box 39695, United Arab Emirates; 9 Private Practice Limited to Prosthodontics and Implant Dentistry, West Vancouver, BC V7V 3K3, Canada; 10 MDRCBB, Minnesota Dental Research Center for Biomaterials and Biomechanics, University of Minnesota, Minneapolis, MN 55455, USA

**Keywords:** dental implants, Immediate Dental Implant Loading, bone transplantation, guided tissue regeneration, periodontal, alveolar ridge preservation

## Abstract

*Background and Objectives:* Immediate implant placement in the esthetic zone presents challenges in maintaining peri-implant tissues due to post-extraction remodeling. Bone grafting has been proposed to support tissue preservation and improve esthetic outcomes. This article reviews the role of grafting in clinical studies and case reports. *Materials and Methods:* A literature search on PubMed and Google Scholar identified studies focusing on immediate implant placement with grafting. The search strategy included articles from 2012 to 2025, in English, from peer-reviewed journals. *Results*: Implant survival is possible without grafting in ideal cases, but grafting is often essential in patients with thin biotypes or esthetic demands. Technique and material selection are critical. The socket shield technique shows promise in preserving buccal tissues despite its complexity. Case reports demonstrated stable soft tissues and favorable esthetic outcomes. *Conclusions*: Grafting should be tailored to the clinical situation. While not always necessary, it is often crucial in compromised sites to ensure long-term esthetic success. Current literature supports predictable outcomes with appropriate grafting protocols.

## 1. Introduction

Immediate implant placement encompasses a variety of techniques, each defined by specific procedural variations tailored to clinical and anatomical circumstances. These approaches are primarily categorized based on three critical clinical decisions: (1) soft tissue management, specifically whether to perform a flapless or flap-based surgical approach; (2) the treatment of the fresh extraction socket, including drilling protocols for achieving primary implant stability; and (3) the timing of provisional restoration placement, also known as immediate provisionalization [1].

The selection of the most appropriate technique is multifactorial, relying on a comprehensive assessment of the patient’s anatomical features, the integrity of the extraction site, and the desired esthetic and functional outcomes [2,3]. The initial decision—whether to perform a flapless procedure or raise a mucoperiosteal flap—is pivotal. A flapless approach preserves the periosteal blood supply and reduces postoperative morbidity, whereas flap elevation offers improved access, visibility, and facilitates bone augmentation when needed [4,5,6]. Following this, achieving primary implant stability involves strategic implant positioning, often by engaging the apical or palatal bone. Grafting materials may be employed to enhance osseointegration and support the peri-implant tissues in cases where a gap remains between the implant and socket walls [7,8].

The decision to place an immediate provisional restoration depends on the quality of primary stability achieved. When sufficient stability is confirmed, immediate provisionalization can help preserve the soft tissue contours and provide instant esthetic benefits. Conversely, a delayed approach may be preferred in situations where initial stability is inadequate, allowing for undisturbed soft tissue healing prior to prosthetic placement [9].

Given the complexity and technique-sensitive nature of these decisions, a comprehensive understanding of each approach is crucial for achieving predictable esthetic and functional success. This paper will therefore provide a narrative review of the current immediate implant placement techniques

## 2. Materials and Methods

**Figure 1:**
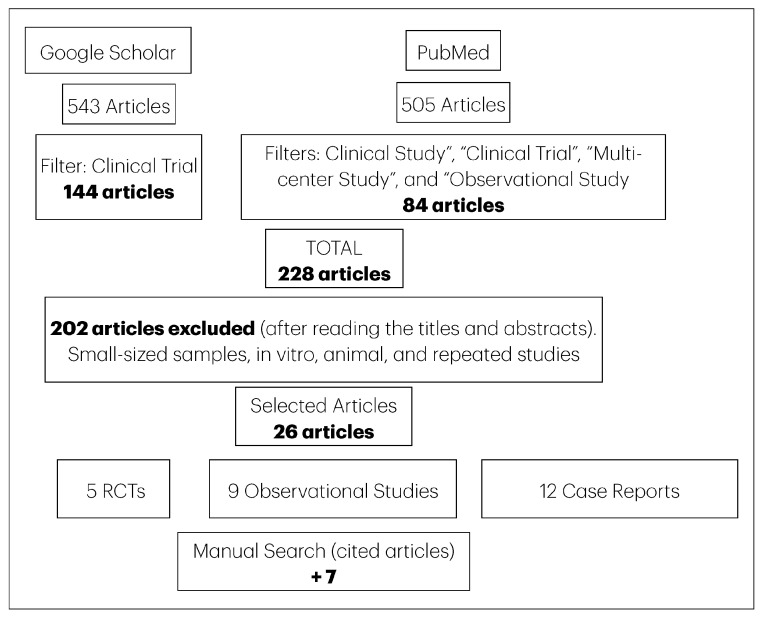
Flowchart illustrating the search strategy and selection process for the articles.

The literature search was performed in two different databases (Google Scholar and PubMed). The following filters were used for Google Scholar: Articles from 2012 to 2025, in English. Systematic reviews, meta-analysis, studies in animals (canine, rats, and rabbits were excluded), and the search strategy was the following: “immediate implant placement” AND “bone grafting”–“systematic review”–meta-analysis–animal–canine–rat–rabbit. The search strategy for PubMed was as follows: (“Immediate Dental Implant Loading” (Mesh)) OR “immediate implant placement” (tiab) OR “immediate implantation” (tiab) OR “post-extraction implant” (tiab) OR “fresh socket” (tiab)) AND (“Bone Transplantation” (Mesh) OR “Alveolar Ridge Augmentation” (Mesh)) OR “bone graft” (tiab) OR “bone grafting” (tiab) OR “socket grafting” (tiab) OR “ridge preservation” (tiab) OR “guided bone regeneration” (tiab)) NOT (“systematic review” (Publication Type) OR “meta-analysis” (Publication Type) OR (animals (MeSH) NOT humans (MeSH))). The search excluded systematic reviews and meta-analyses, in vitro studies, and studies in animals or in vitro and ex vivo studies. A manual search was made from the cited articles and found other 14 articles. The details of the search and selection of the articles are shown in Figure 1.

Two independent reviewers were involved in screening and data extraction. The research question was “Does immediate implant placement with bone augmentation in post-extraction sockets with buccal bony defects of ≥5 mm in the esthetic zone lead to comparable or superior peri-implant hard and soft tissue outcomes, esthetics, and patient satisfaction over a 5-year period, compared to delayed implant placement after alveolar ridge preservation?” This research question compares two non-randomized interventions (immediate vs. delayed implant placement) for which a randomized controlled trial (RCT) would be unethical or impractical.

### Confounding Factors

Initial Bone and Tissue Conditions: The baseline state of the patient’s bone and soft tissue is a significant confounder. Differences in buccal bone thickness, the size and type of the bony defect, and the atient’s gingival tissue phenotype (biotype) could all affect the outcome, regardless of the intervention chosen. For example, a thicker gingival biotype is associated with more favorable esthetic outcomes and less recession.

Patient Health and Habits: Systemic health and patient habits can influence healing and long-term implant success. Factors such as uncontrolled diabetes, smoking (especially heavy smoking more than 20 cigarettes per day), and parafunctional habits like bruxism are all known to negatively impact treatment outcomes.

Surgical Technique and Operator Experience: The skill and experience of the surgeon affects the result, particularly for a technically sensitive procedure like immediate implant placement. Key aspects include the atraumatic extraction of the tooth to preserve bone, the proper positioning of the implant, and the careful execution of bone grafting and soft tissue procedures. A flapless approach versus a flap elevation is another technical difference that could influence outcomes.

Implant and Grafting Material Characteristics: The type of implant used, including its design (e.g., tapered, platform-switched, micro threaded surface), length, and diameter, can influence primary stability and long-term bone maintenance. The choice of bone grafting material (e.g., xenograft, allograft) and whether a membrane is used also plays a role in bone regeneration and stability.

Prosthetic Protocol: How and when the definitive restoration is placed can affect the final soft and hard tissue contours. The timing of provisionalization, the type of restoration (e.g., screw-retained vs. cement-retained), and the occlusal load on the implant can all be confounding factors. For instance, excess cement in a cement-retained restoration can lead to bone loss and inflammation.

## 3. Results

### Comprehensive Review Outcome

The risk of bias (RoB) analysis for a selection of clinical studies on immediate implant placement was made using Cochrane Risk of Bias (ROBINS-I), used for Randomized Controlled Trials (RCTs). The purpose of this analysis is to evaluate the methodological quality of each study and determine the level of confidence in their reported outcomes.

Table 1 summarizes the study characteristics of observational studies from 2012 to 2025 on immediate implant placement studies.

Table 2 describes the overall risk of bias for each study and provides a brief justification for the assessment, highlighting the key factors that influence each study’s internal validity.

An analysis of the ‘Risk of Bias’ column reveals a prevalence of ‘Low risk’ classifications in 9 out of 13 studies. Conversely, only 4 studies were categorized as having a ‘Moderate risk’ of bias. This distribution suggests a general adherence to robust methodological practices within the reviewed studies, aimed at minimizing inherent biases. Based on the “Random Sequence Generation” column in the provided data, a few studies used sophisticated computer-generated randomization lists [20,21,22,23]. One single study indicated random assignment without explicitly detailing the generation method, which mentions that patients were “randomly assigned” but does not explicitly state the method beyond the computer-generated list for allocation [20]. Two other studies used simple random sequences [21,22], while another used block randomization [23]. Another study used a more rudimentary manual technique—a shuffled deck of cards [24].

## 4. Discussion

### 4.1. The Role of Grafting in Hard and Soft Tissue Stability

The rationale for bone grafting during immediate implant placement extends beyond simply filling a void; it is intended to actively influence the healing of both hard and soft tissues to ensure long-term stability and esthetics. The type of graft material and the patient’s pre-existing tissue quality are significant variables that affect the outcome. The interaction between the graft, the implant, and the surrounding biological environment determines whether the tissue volume is maintained, enhanced, or diminished over time.

Observational studies have shed light on how grafting influences tissue healing and dimensional changes. One prospective clinical study found that a history of bone grafting prior to implant placement can positively affect subsequent soft tissue healing [11]. These results suggest augmented sites provide a more favorable environment for peri-implant tissue health. The choice of graft material also plays a role, as a clinical study assessing different bone grafts in immediate implant sites found variations in implant stability, indicating that material properties can influence the osseointegration process [12]. In cases with a thin buccal plate in the anterior maxilla, a case series demonstrated that immediate implant placement with simultaneous buccal bone augmentation led to favorable outcomes at one year, with stable bone and ridge dimensions [13].

These studies underscore the importance of a comprehensive site assessment before deciding on a grafting strategy. In sites with bone loss or a thin buccal plate, not grafting can cause facial recession and gray tissue discoloration from the implant. A case series on immediate implants with facial bone dehiscence found that while the procedure can be successful, the correlation between pre-operative dehiscence and postoperative gingival recession highlights the inherent risks [14]. Therefore, grafting should be viewed not only as corrective but as a proactive step to strengthen the tissue biotype and ensure a stable foundation for the final restoration.

### 4.2. Immediate Provisionalization and Grafting in the Esthetic Zone

The decision to place an immediate provisional restoration and to graft the peri-implant gap is a critical step in achieving predictable esthetic outcomes [15,16]. This approach is particularly relevant in the esthetic zone, where the preservation of soft tissue architecture is important. The literature provides several examples of high success rates when combining these techniques, though the protocols and materials vary. The primary goals are to support the facial tissues, prevent gingival recession, and maintain the natural emergence profile of the restoration from the outset of treatment [17].

Evidence from prospective case series supports the viability of this combined approach. In 110 implants placed without graft material (selected based on sufficient bone beyond the root apex and absence of pathology), survival over 5 years was very high, with only five failures [18]. These results demonstrate predictability. A study involving 27 patients who received immediately placed and provisionalized implants with bone grafting in the esthetic zone reported predictable success and survival, highlighting the importance of 3D implant positioning and a non-functional, screw-retained provisional [19]. Similarly, another case series involving 15 patients who received immediate implants with provisional restoration demonstrated stable changes in hard and soft tissues over a 23.2-month follow-up [19]. These results reinforce the technique’s reliability when strict clinical protocols are followed. Furthermore, a study focusing on peri-implant bone response after immediate placement and provisionalization also found favorable outcomes, suggesting this protocol can maintain bone levels effectively in the esthetic zone [20].

These findings suggest that, when primary stability is achieved, immediate provisionalization with bone grafting is an effective strategy for managing peri-implant tissues. The provisional crown acts as a scaffold for the healing gingiva, while the bone graft helps to fill the residual gap and support the facial bone plate, mitigating the risk of resorption and subsequent tissue collapse. However, as noted in a retrospective study analyzing treatment for labial soft tissue recession, the use of mineralized allograft for guided bone regeneration can be an effective method to address and prevent such esthetic complications, emphasizing that grafting is a key component for long-term tissue stability [21,22].

### 4.3. Socket Shield Technique as an Alternative to Complete Extraction

An alternative approach to conventional immediate implant placement is the socket shield technique (SST), which involves retaining the buccal portion of the root to preserve the periodontal ligament and, consequently, the buccal bone plate. This technique was developed to counteract the physiological bone remodeling and resorption that typically follows tooth extraction. By leaving the labial root fragment in situ, the blood supply from the periodontal ligament is maintained, which is thought to provide a more stable biological environment for the peri-implant tissues [23].

Despite its potential benefits, the socket shield technique requires meticulous execution and is associated with a steep learning curve. The clinician must be proficient in sectioning the root without damaging the buccal plate and placing the implant in the correct three-dimensional position relative to the retained root fragment. Complications such as infection, failure of the shield to integrate, or exposure of the shield can occur. Therefore, although SST provides a strong biological rationale for ridge preservation, its use should be limited to experienced clinicians and carefully selected cases where the benefits outweigh the risks [24].

### 4.4. Insights from Randomized Clinical Evidence

RCTs provide the highest level of evidence for evaluating clinical interventions. Several RCTs have investigated the efficacy of bone grafting in immediate implant placement. These studies offer more definitive insights into whether grafting provides a clinically significant benefit compared to non-grafted sites. The evidence, however, is not entirely conclusive and often depends on the specific clinical scenario, outcome measures, and follow-up duration.

In contrast, a different randomized trial employing “dual zone grafting” with a mix of autogenous and xenogeneic bone reported that this technique significantly improved tissue volume and esthetic outcomes. This discrepancy suggests that the grafting technique and material choice are critical variables [28].

One RCT evaluating grafting in sites with a thin labial plate found that adding a xenograft yielded only a minimal, statistically insignificant increase in horizontal bone width after one year compared with ungrafted sites [29]. One RCT comparing immediate versus early implant placement in the esthetic area found that both treatments can yield successful outcomes, suggesting that clinicians have some flexibility in timing, though immediate placement offers the benefit of a shorter treatment period [31,32,33]. Grafting during immediate placement may offer minor improvements in horizontal gap fill and soft-tissue esthetics in the anterior zone, but evidence of significant volumetric preservation is inconsistent (~0.2 mm difference).

### 4.5. Limitations of the Review

This narrative review has several limitations. The search strategy was not as exhaustive as that of a systematic review and was limited to two databases, which may have resulted in the omission of some relevant studies. The heterogeneity among the included studies in terms of methodology, follow-up duration, and outcome measures makes direct comparisons difficult and limits the ability to draw definitive, overarching conclusions. The constraints here outlined directly qualify the strength of the discussion’s conclusions, revealing the unreliable foundation of the evidence. The discussion uses a limited, non-exhaustive selection of studies, which means its inferences may be based on incomplete information. This raises the possibility that other studies not included in the review could contradict or significantly alter the findings, making the conclusions about the benefits of grafting tentative rather than definitive. For example, while the text mentions “high success rates,” this observation might be skewed if a broader, more representative sample of the literature were examined.

The heterogeneity of the included studies further undermines the reliability of the discussion’s inferences. The studies referenced varied widely in their methods, follow-up times, and outcome measures, making direct comparisons difficult. This inconsistency makes it impossible to draw a single, strong conclusion about grafting’s effectiveness. The inference that the grafting technique and material are critical variables is a logical one, but it remains a qualified statement because the underlying evidence is too inconsistent to support a definitive conclusion.

## 5. Conclusions

Integrating bone grafting into immediate implant placement protocols is beneficial for achieving predictable and stable aesthetic outcomes, especially in the challenging anterior maxilla. Although implants may survive in certain cases without grafting, the evidence suggests that bone grafts help preserve alveolar bone dimensions and support peri-implant soft tissues. This is critical for long-term success. The chosen technique—whether flapped or flapless, the type of graft material, and the use of immediate provisionalization—must be customized to the patient’s specific anatomical and clinical needs. A comprehensive approach that combines digital planning, atraumatic surgery, soft tissue augmentation, and precise prosthetic management is essential for success in complex aesthetic cases.

## Figures and Tables

**Table 1:** Study characteristics of selected observational studies (2012 to 2025) on immediate implant placement studies [10,11,12,13,14,15,16,17,18,19].

Author and Year	Study Design	Follow-Up	Primary Outcomes
Amato et al., 2022 [10]	Observational study	2 to 5 years ($\bar{X}$ 3.5 years)	100% implant survival rate. $\bar{X}$ MBL 0.43 ± 0.23 mm. Mean coronal gingival level gained 1.6 ± 1.17 mm and mean vertical alveolar bone gained 4.0 ± 1.5 mm. The average Pink Esthetic Score was 12.5.
Atalay et al., 2013 [18]	Retrospective clinical trial	5 years	95.5% implant survival rate. No statistically significant correlation was found between implant failure and the reason for extraction or implant location. A success rate of 95.3% for implants in infected sockets was reported.
Blanco et al., 2025 [6]	Narrative review with a systematic search strategy	NS.	The existing literature suggests that autologous platelet concentrates (APCs) provide a clear benefit in alveolar bone augmentation, leading to additional bone gain and improved wound healing. Vertical bone augmentation is less predictable and has a higher complication rate than the horizontal approach.
Chen et al., 2021 [13]	Prospective case series	1 year follow-up	Simultaneous buccal bone augmentation maintained predictable buccal bone thickness in sites with a thin buccal plate (<1 mm). Mean buccal bone thickness at 2 mm apical to the implant-abutment junction was 2.52 mm at the 1-year follow-up. Overall ridge width remained stable from before surgery to the 1-year follow-up.
De Angelis et al., 2021 [3]	Narrative review	NS.	The socket-shield technique (SST) is a promising strategy for immediate dental implant placement, with a cumulative survival rate of 98.25%. It helps to preserve buccal bone volume and soft tissues, meeting aesthetic demands.
Jalaluddin et al., 2021 [12]	Clinical study	12 months	Both xenograft and freeze-dried bone allograft improved implant stability and marginal bone levels. No significant difference was found between the two grafting materials.
Jebelli et al., 2024 [7]	Narrative review	NS.	The review highlights the potential of bioactive materials and growth factor delivery systems, such as BMPs and VEGF, to enhance osseointegration and accelerate bone regeneration. In vivo studies show improved osseointegration with these techniques compared to traditional methods.
Khzam et al., 2014 [17]	Clinical case series	12 to 37 months (mean 23.2 ± 7.6 months)	100% implant survival with no complications. Mean mesial bone gain of 1.20 ± 1.01 mm and distal bone gain of 0.80 ± 1.14 mm were observed. Soft tissue changes were not statistically significant.
Kofina et al., 2021 [11]	Prospective clinical observation	4 months	Healing in grafted sites showed delayed wound closure and persistent ischemia. Grafted sites had greater buccal bone thickness changes compared to non-grafted sites. The history of bone grafting altered the clinical, physiological, and molecular healing response.
Le et al., 2016 [15]	Retrospective clinical case series	Approximately 1 year	Use of mineralized allograft and a resorbable membrane increased alveolar hard and soft tissue dimensions. Mean crestal bone thickness increased by 1.84 mm and mean soft tissue thickness increased by 1.28 mm.
Levin and Wilk, 2013 [19]	Prospective case series	Minimum 12 months of definitive restoration loading	100% implant survival was achieved. Bone maintenance (BM) was noted in 86% of cases. All patients were satisfied with the esthetic outcome.
Liñares et al., 2023 [4]	Critical review	NS.	Bone grafting benefits are more apparent in the anterior maxilla with thin buccal bone plates. Flapless procedures and connective tissue grafts lead to less bone loss and improved aesthetic outcomes. The use of membranes in intact sockets does not seem to improve clinical outcomes.
Martin et al., 2015 [16]	Case series study	8 months	Bone loss occurred on the mesial bone crest and buccal face. Bone deposition was observed where the bone met the implant surface on the mesial and distal faces. Buccal bone wall width showed statistically significant bone loss at all measured levels.
Meng et al., 2021 [20]	Case report	6.5 years	100% success and survival rates were achieved. Stable hard and soft tissue levels were maintained. The patient was satisfied with the aesthetic outcome throughout the follow-up period.
Milanovic et al., 2021 [2]	Narrative review, CBCT study	Not applicable	The possibility for immediate implant placement can be affected by the shape of the nasopalatine canal (NPC). The presence of accessory canals (ACs) may increase complications. Interradicular septum (IRS) characteristics are important criteria for choosing implant properties for successful immediate implant placement.
Mizuno et al., 2022 [14]	Case series	1 year after definitive restoration	100% implant survival rate. A positive correlation was found between the width and depth of facial alveolar bone dehiscence and the degree of gingival recession.
Nobre et al., 2023 [9]	Retrospective clinical study	14 years clinical, 10 years radiographic	A cumulative implant survival rate of 93% and a success rate of 88.1% were reported. Average marginal bone loss was 2.01 mm at 10 years. Smoking was found to significantly increase the incidence of biological complications.
Sanz-Sánchez et al., 2022 [5]	Narrative review	NS.	A classification for complications in bone grafting was provided, including soft tissue dehiscence, infections, bone fractures, and neural damage. Vertical bone augmentation has a lower predictability and a higher complication rate than horizontal augmentation.
Sghaireen et al., 2023 [8]	Narrative review	NS.	Autogenous grafts are the “gold standard” for bone reconstruction due to their osteogenic, osteoinductive, and osteoconductive properties. Proper graft handling, preparation of the recipient site, and soft-tissue coverage are crucial for successful grafting.
Vasiljevic et al., 2024 [2]	Narrative review, CBCT study	NS.	Immediate implant placement may be affected by nasopalatine canal (NPC) shape in the anterior maxilla. The presence of accessory canals (ACs) may increase complications. Interradicular septum (IRS) characteristics are important for choosing implant properties.

NS: Not Specified. MBL: marginal bone loss.

**Table 2:** Risk of bias analysis of selected RCTs (2012 to 2025) on immediate implant placement studies [20,21,22,23,24,25,26,27,28,29,30].

Author, Year	Findings	Potential for Bias	Confounding Factors Mentioned	Risk of Bias
Cardaropoli et al., 2014 [20]	Grafted group: horizontal ridge resorption (0.69 ± 0.68 mm vs. 1.92 ± 1.02 mm) vertical loss (0.58 ± 0.77 mm vs. 1.69 ± 1.74 mm) at 12 months. 100% and 96.15% implant survival rates for test and control groups.	Limitation: all sites had intact bone walls, which may limit the generalizability of the findings.	Patient age, sex, smoking habits (heavy smokers excluded), and systemic health.	Low
Mastrangelo et al., 2018 [21]	NSD in MBL changes or PD between the grafted and non-grafted groups after 3 years. PES and patient satisfaction significantly higher in the grafted group.	Clinicians not blinded (potential performance bias).	Age, sex, smoking habits, history of periodontitis, implant size, and bone quality.	Moderate
Guadilla et al., 2022 [22]	Autologous bone, with/without PRGF, highest short term (2–4 months) mineralization. PRGF alone may interfere with normal bone healing compared to spontaneous healing.	Split-mouth RCT. Unequal sample sizes and follow-up periods across groups.	Smoking status, age, sex, and cause of extraction.	Moderate
Gurbuz & Ceylan, 2025 [23]	KMW and PES significantly greater in the non-grafted SST group after 1 year. Hard tissue changes similar between the SST and GBR groups.	Surgeon was not blinded to the group assignment (performance bias).	Age, sex, ASA health status, smoking habits, periodontal health, and the presence of root fractures or infection.	Low
Oliveira et al., 2021 [24]	SST resulted in better preservation of the buccal-to-palatal crest distance and buccal wall height compared to minimally traumatic extraction. Preservation of buccal wall thickness was lower in the SST group.	Small sample size and short follow-up period.	Age, sex, tooth position, and patient systemic health were controlled. Patients with infection, buccal dehiscence, or who were smokers were excluded.	Low
Happe et al., 2022 [25]	ADM showed no difference in soft tissue volume change compared to an autogenous connective tissue graft (CTG) after 1 year. The ADM group reported significantly less postoperative pain.	Small sample size and 1-year follow-up.	Age, sex, smoking habits (>10 cigarettes/day excluded), periodontal status, and buccal plate condition.	Low
Perez et al., 2020 [26]	The customized healing abutment group showed significantly higher Papilla Index scores at 4 and 12 months than the standard abutment group. Mesial bone loss was significantly higher in the standard group after 12 months.	Groups were not balanced for confounding factors like age and smoking. Small sample size and 1 year follow-up.	Age, gender, smoking habits, reason for tooth extraction, implant position, and insertion torque value.	Moderate
Girlanda et al., 2019 [27]	The test group (with bone graft) showed higher soft tissue height at mesiobuccal and distobuccal sites at 3 and 6 months compared to the control group (without biomaterial). The test group also showed higher buccolingual ridge dimension at 6 months.	Small sample size and short 6-month follow-up.	Systemic diseases, medications, smoking, previous grafts, and insufficient bone were exclusion criteria. The study also required a present buccal alveolar wall and absence of acute infection.	Low
Bajaj et al., 2025 [28]	The test group (with CGF-enriched bone graft) showed significantly better outcomes in ridge width, vertical distance, and jump space compared to the control group. The esthetic score (TS) was significantly improved in the test group. Both groups had high patient satisfaction and no implant loss during the 12-month study.	Short 12-month follow-up.	Systemic health, history of irradiation, use of bisphosphonates, smoking, poor oral hygiene, and parafunctional habits were exclusion criteria. Intact alveolar bone walls and sufficient bone for primary stability were required.	Low
Hamed et al., 2023 [29]	The particulate bone graft (MinerOss X) significantly increased facial bone thickness (FBT) compared to the collagen plug and DBM-Grafton groups after 12 months. All groups showed high PES scores (>10) with no significant difference between them.	Sequentially numbered, opaque, sealed envelopes (SNOSE) for allocation concealment, reducing selection bias. Small sample size and short follow-up period.	Non-restorable teeth or roots without infection, sufficient apical and palatal bone, good systemic health, and compliance were included. Heavy smokers and medically compromised patients were excluded.	Low
El Zahwy et al., 2019 [30]	The inlay technique resulted in a statistically significant vertical bone gain (3.34 mm) and less crestal bone loss (1.65 mm) compared to the onlay group (−0.02 mm gain and 4.77 mm loss). The onlay group had a higher rate of complications, including wound dehiscence and graft loss.	A randomized comparative clinical study with randomization via computer software. The study has a short 6-month follow-up period. The high rate of complications in the onlay group could introduce bias.	Patients free from systemic disease, prior grafting, or local pathosis. Edentulous ridges with specific vertical and horizontal dimensions were included.	Low
Wanis et al., 2022 [31]	No significant difference in PES, MFR, STT, or KTW was found between the dual-zone (DZ) and bone-to-crest (BCG) groups after 12 months. BBL was not prevented in either group, despite bone graft use. The DZ group had a statistically significant increase in post-operative swelling.	This is a randomized controlled trial with blinded outcome assessors, data analysts, and participants, which minimizes bias. The surgeon was not blinded.	Patient age (20–50), systemic health, smoking status, and parafunctional habits were exclusion criteria. The study required an intact buccal bone of at least 1 mm thickness.	Low
Meijer et al., 2025 [32]	After 10 years, the mean marginal bone level change was not statistically different between the immediate and delayed groups. Buccal bone thickness, clinical outcomes, esthetics, and patient satisfaction were also not statistically different. A low dropout rate and consistent outcomes suggest the procedures are predictable and stable long-term.	Initial sample size calculation did not fully met due to dropouts. The post hoc power calculation revealed a lower-than-intended power, increasing the risk of false-negative findings.	Patient age, sex, and the presence of a buccal bony defect ≥5 mm. Patients with signs of infection and required sufficient palatal bone for primary stability were excluded.	Moderate

NSD: No statistically significant differences: MBL: marginal bone level; PD: probing depth; PES: Pink Esthetic Score; KMW: Keratinized mucosa width; SST: socket shield technique; ADM: acellular dermal matrix.

## Data Availability

Not applicable.
